# Subgenome-biased expression and functional diversification of a Na^+^/H^+^ antiporter homoeologs in salt tolerance of polyploid wheat

**DOI:** 10.3389/fpls.2022.1072009

**Published:** 2022-12-07

**Authors:** Mei Zheng, Jinpeng Li, Chaowu Zeng, Xingbei Liu, Wei Chu, Jingchen Lin, Fengzhi Wang, Weiwei Wang, Weilong Guo, Mingming Xin, Yingyin Yao, Huiru Peng, Zhongfu Ni, Qixin Sun, Zhaorong Hu

**Affiliations:** ^1^ Frontiers Science Center for Molecular Design Breeding/Key Laboratory of Crop Heterosis and Utilization (Ministry of Education), Beijing Key Laboratory of Crop Genetic Improvement, China Agricultural University, Beijing, China; ^2^ Institute of Crop Sciences, Xinjiang Academy of Agricultural Sciences, Urumuqi, China; ^3^ Hebei Key Laboratory of Crop Salt-alkali Stress Tolerance Evaluation and Genetic Improvement, Cangzhou Academy of Agriculture and Forestry Science, Cangzhou, China

**Keywords:** adaptability, polyploid, wheat, salt tolerace, homoeolog, expression divergence

## Abstract

Common wheat (*Triticum aestivum*, BBAADD) is an allohexaploid species combines the D genome from *Ae. tauschii* and with the AB genomes from tetraploid wheat (*Triticum turgidum*). Compared with tetraploid wheat, hexaploid wheat has wide-ranging adaptability to environmental adversity such as salt stress. However, little is known about the molecular basis underlying this trait. The plasma membrane Na^+^/H^+^ transporter Salt Overly Sensitive 1 (SOS1) is a key determinant of salt tolerance in plants. Here we show that the upregulation of *TaSOS1* expression is positively correlated with salt tolerance variation in polyploid wheat. Furthermore, both transcriptional analysis and GUS staining on transgenic plants indicated *TaSOS1-A* and *TaSOS1-B* exhibited higher basal expression in roots and leaves in normal conditions and further up-regulated under salt stress; while *TaSOS1-D* showed markedly lower expression in roots and leaves under normal conditions, but significant up-regulated in roots but not leaves under salt stress. Moreover, transgenic studies in Arabidopsis demonstrate that three *TaSOS1* homoeologs display different contribution to salt tolerance and *TaSOS1-D* plays the prominent role in salt stress. Our findings provide insights into the subgenomic homoeologs variation potential to broad adaptability of natural polyploidy wheat, which might effective for genetic improvement of salinity tolerance in wheat and other crops.

## Introduction

Allopolyploidization played a significant role in the speciation and evolution of both wild and cultivated plants (Jiao et al., 2011; Hegarty et al., 2013). Allopolyploid organisms often outperform their ancestral species in several aspects, such as increased vigor and potential adaptation to a wider range of environmental conditions (Otto, 2007; Madlung, 2013; Wu et al., 2020). As an allohexaploid species, common wheat (*Triticum aestivum*, BBAADD) is originated through hybridization and chromosome doubling of three ancestral diploid species, related to nowadays existing *Triticum urartu* (AA), *Aegilops speltoides* (SS), and *Ae. tauschii* (DD) (Goncharov, 2011). Thus, most of the genes present in common wheat as triplicate homoeologs derived from the ancestral species (Feldman and Levy, 2005; IWGSC et al., 2018). Hexaploid wheat presents broader adaptability to different environments and increasing the tolerance to both biotic and abiotic stresses compared with tetraploid wheat (Dubcovsky and Dvorak, 2007; Feldman et al., 2012; Pallotta et al., 2014; Yang et al., 2014; Yang et al., 2018). Research increasingly suggests that the differential expression and functional diversification of homoeologous genes plays an important role in the evolutionary success of polyploidy wheat species (Shitsukawa et al., 2007; Hu et al., 2013; Li et al., 2014; Yang et al., 2014; Uauy, 2017; Wang et al., 2018). Nevertheless, our understanding of the molecular basis underlying of these traits or the relative contributions of allohexaploidization on the trait development are still limited in hexaploid wheat. Exploring the mechanisms of evolutionary genetic changes or that modulating the expression of triplicate homoeologs may provide clues regarding the origin of important traits that have arisen during the evolution and domestication of allopolyploid wheat (Borrill et al., 2015; Ding and Chen, 2018).

Salinity is considered as one of the most severe environmental hazards for wheat growth and grain yield worldwide (Munns and Tester, 2008). Salinity stress can decrease wheat growth and productivity by reducing water uptake and causing nutrient disorders and ion toxicity in many regions (Huang et al., 2006; Byrt et al., 2014; Munns and Gilliham, 2015). Therefore, understanding the mechanisms of response and adaptation to salt stress and then improving the salinity tolerance of crops are main targets for future researches. It is well known that hexaploid bread wheat is generally more salt tolerant than its tetraploid wheat progenitor (Dvorak et al., 1994; Munns et al., 2012; Yang et al., 2014). In saline environments, hexaploid wheat has a low rate of Na^+^ transport to the shoot and maintains a high ratio of K^+^ to Na^+^ in leaves than durum wheat, this trait is conferred, at least partially, by the *Kna1* locus that mapped to the distal region of chromosome 4DL (Gorham et al., 1987; Dubcovsky et al., 1996). Further studies indicated that gene *TaHKT1;5-D* could be as the candidate gene of *Kna1* locus, encodes a Na^+^-selective transporter located on the plasma membrane of stelar cells in roots of wheat, confers the essential salinity tolerance in bread wheat *via* shoot Na^+^ exclusion (Byrt et al., 2014). The tetraploid wheat (genome BBAA) does not exclude Na^+^ to the same extent as bread wheat, since the A subgenome of tetraploid and hexaploid wheats, which was contributed by *Triticum urartu* (A^u^A^u^), lacks the *HKT1;5* gene; the *HKT1;5-B*, albeit present, may have lost its function for salt tolerance (James et al., 2006).

Besides the HKT-type transporters confer salinity tolerance by promoting sodium exclusion (Ren et al., 2005; Huang et al., 2008), the salt-overly-sensitive (SOS) signal transduction pathway is to be widely regarded as important mechanism for maintaining intracellular ion homeostasis and salt tolerance in plants (Zhu, 2003; Du et al., 2011). This pathway including three key components (SOS1, SOS2 and SOS3). SOS3 encodes a myristoylated Ca^2+^ -binding protein that capable of sensing Ca^2+^ oscillations elicited by salt stress and is able to activate and recruit the serine/threonine protein kinase SOS2 to the plasma membrane to achieve phosphorylation and activation of SOS1, a Na^+^/H^+^ antiporter, thus causes efflux of excessive Na^+^ from the cell (Shi et al., 2000; Qiu et al., 2002; Guo et al., 2004; Quintero et al., 2011; Ji et al., 2013). Compared with *sos2* and *sos3* mutant plants, *sos1* mutant plants are even more sensitive to NaCl treatments (Wu et al., 1996). In Arabidopsis, *SOS1* preferentially express in epidermal cells at the root tip and in parenchyma cells at the xylem/symplast boundary of roots, stems, and leaves (Shi et al., 2002; Mahi et al., 2019). Under salt condition, the xylem sap of *sos1* mutant plants contain more Na^+^ suggested that SOS1 is crucial for controlling long-distance Na^+^ transport from root to shoot (Shi et al., 2002). Increased SOS1 activity would also lead to apoplastic alkalinization and cytoplasmic acidification which might serve as a signal to mediate gene regulation (Chung et al., 2008). Recently, the importance of SOS1-facilitated Na^+^ flux in the salt tolerance of rice, soybean, tomato and cotton were analyzed, suggesting that SOS1 is conserved in higher plants including both monocots and dicots (Olías et al., 2009; Che et al., 2019; Mahi et al., 2019). In wheat, previous studies also indicated expression of *TaSOS1* can enhance the salt tolerance of a yeast strain lacking the major Na^+^ efflux systems and improves the growth and salt tolerance of transgenic *Arabidopsis* and tobacco (Xu et al., 2008; Feki et al., 2014; Zhou et al., 2016). However, little is known about the relative contributions of *TaSOS1* homoeologs to salt tolerance in polyploid wheat. In addition, there are currently no comparative analysis focusing on assessment of genome-specific expression and functional variances among *TaSOS1* homoeologous genes in allopolyploid wheat. Except that Feki et al. found the promoter of *TaSOS1-AB* and *TaSOS1-D* are age-dependent and *TaSOS1-AB* is abiotic stress-inducible at different developmental stages (Feki et al., 2015).

In this study, we show that three Na^+^/H^+^ antiporter *TaSOS1* homoeologs of wheat exhibited subgenome-biased expression and functional diversification in response to salt stress and among three *TaSOS1* homoeologs, *TaSOS1-D* displays the prominent contribution to salt tolerance in transgenic *Arabidopsis*. Our findings provide insights into the subgenomic homoeologs variation potential to facilitate the wide-ranging adaptability of natural hexaploid wheat and other polyploidy species.

## Materials and methods

### Plant materials and growth conditions

Two set of synthetic allohexaploid wheat (SCAUP/SQ523, DM4/Y199) and their tetraploid (SCAUP, DM4) and diploid (SQ523, Y199) progenitors, 48 natural wheat accessions with different ploidy levels (**Table S1**) were used to examine the difference of salt tolerance between different ploidy wheats and to explore the expression pattern of *TaSOS1* before and after salt treatment. wheat plants were grown under 16/8 h, light/dark conditions at a temperature of 22°C. wheat variety Nongda3338 was used to clone the three CDS and promoter sequences of *TaSOS1* homoeologous genes and analyze the expression pattern of three *TaSOS1* homoeologous genes under normal or salt condition.

Arabidopsis (*Arabidopsis thaliana*) wild-type Columbia was used as the recipient of the overexpression and promoter analysis of *pSOS1:uidA* reporters transformation. Sterilized seeds were incubated at 4°C for 3 d then sowed on MS plates containing 1% (w/v) Suc and 0.8% (w/v) agar. Seedlings were grown under a 16-h-light/8-h-dark cycle at 22°C in a growth room.

### Salt tolerance assays

For salt tolerance comparisons, seedlings of the synthetic allohexaploid wheat genotype (SCAUP/SQ523), and their respective tetraploid and diploid parents, 48 natural wheat accessions with different ploidy levels were grown in vermiculite and watered with half strength Hoagland’s liquid medium (pH 6.0). The seedlings were treated with 0 or 200 mM NaCl at three-leaf stage for 10 d. Then the chlorophyll content, shoot fresh weight, root length and root fresh weight were quantified and photos were taken. Root length was quantified by measurement with a ruler and the fresh mass of the shoots and roots were measured using an automated electronic scale. Chlorophyll were measured by aqueous acetone (80%) described earlier.

For *Arabidopsis* germination rate analysis, Seeds were sown on MS medium supplemented with 0, 100, 200 mM NaCl, and germination rates were scored every 24 hours. For the salt tolerance assay of plants grown in soil, 5-d-old seedlings were transferred from MS medium to soil. The seedling of wild type and transgenic lines plants grown in soil pots were irrigated with 250 mM NaCl every 5 d, then the chlorophyll content, shoot fresh weight and the leaves electrolyte leakage of each genotype were evaluated after 15 d. For whole growth duration salt tolerance assay, seedlings plants were irrigated every 5 d with or without the addition of 200 mM NaCl until seed maturation.

### Genomic DNA and total RNA extraction

The genomic DNA of wheat genotype Nongda3338 was extracted using the CTAB protocol. Total RNA was extracted with TRIzol reagent (Invitrogen), and purified RNA was treated with DNase I. Subsequently, First-strand cDNA synthesis was performed using HiScipt II One Step RT-PCR Kit (Vazyme Biotech, Nanjing, China).

### Quantitative RT-PCR

Real-time qRT-PCR analysis was conducted using SYBR Premix EX TagTM (TaKaRa, Dalian, China) in a volume of 20μl in a Bio-Rad CFX96 real-time PCR detection system3. The PCR parameters were as follows: 94°C for 3 min, followed by 40 cycles of 94°C for 15 s, 60°C for 20 s, and 72°C for 20 s. Each sample was quantified at least in triplicate and normalized using *Actin* as an internal control.

### Gene cloning and vector construction

Specific primer sets were designed to obtain the CDS and promoter sequences of *TaSOS1-A*, *TaSOS1-B* and *TaSOS1-D*. The PCR was performed using the Tks Gflex DNA Polymerase (TaRaKa, Beijing, China) and the PCR products were recovered by TIANgel Midi Purification Kit (TIANGEN BIOTECH, Beijing, China) then cloned into the pEASY-Blunt Simple Cloning vector (TransGen Biotech, Beijing, China) and sequenced. Amplified fragments of the full-length coding sequence of *TaSOS1-A*, *TaSOS1-B* and *TaSOS1-D* were cloned into the binary expression vector pCAMBIA1300 (driven by the CaMV 35S promoter). The promoter region of target genes that contains about 2-kb fragment upstream of the coding sequence was fused to the reporter gene encoding GUS and was then cloned into the binary expression vector pCAMBIA1300 to generate reporters. All the constructs were constructed by In-Fusion PCR Cloning Kits (TaRaKa, Beijing, China) and were confirmed by sequencing. These vectors were transferred into *Agrobacterium tumefaciens* strain GV3101 and transgenic plants were identified on Murashige and Skoog medium containing hygromycin. The primers employed for plasmid construction are listed in **Table S2**.

### GUS histochemical staining

Two-week-old transgenic *Arabidopsis* seedlings were transferred to MS medium (1% Suc) with 0 or 200 mM NaCl, and after 24h whole seedlings or different tissues expressing the GUS gene were immersed in staining buffer ((100 mM NaPO_4_, 0.5 mM K_3_[Fe(CN)_6_, 0.5 mM K_4_[Fe(CN)_6_], 10 mM EDTA, 1 mg/ml 5-bromo-4-chloro-3-indolyl-b-D-glucuronide (X-gluc)) at 37°C in the dark for overnight. Subsequently, samples were washed in 75% ethanol and the intensity of color development in different tissues was monitored and photographed.

### Ion measurements

For measurement of Na^+^ and K^+^ contents in the seedling leaves, 10-day-old uniform seedlings were transferred to MS plates with 100 mM NaCl solution. 2 days later, the samples were harvested and dried, then dissolved in HNO_3_ and analyzed by inductively coupled plasma optical emission spectrometry (FP6410, INESA).

## Results

### Sequence analysis of *TaSOS1* homoeologs in hexaploid wheat

**Figure 1:**
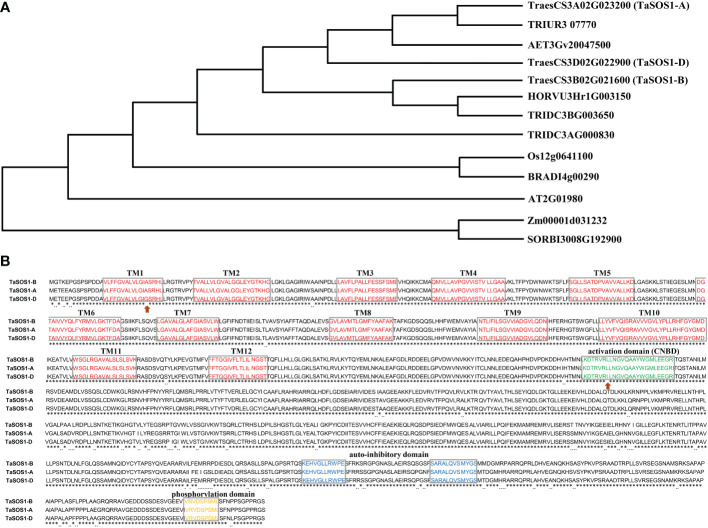
Sequence analysis of three *TaSOS1* homoeologous genes. **(A)** Phylogenetic relationship analysis of three TaSOS1 homoeologs with other members in multiple species. The accession numbers of selected species and gene IDs are listed as **Table S3**. **(B)** Comparison of amino acids in three TaSOS1 homoeologs. Identical amino acids are indicated by asterisks, gray boxes indicate conserved domains among the three proteins, and the red arrows indicate amino acid difference within the conserved domains among three TaSOS1 homoeologs.

In order to identify *TaSOS1* homoeologous genes in wheat, we conducted a BLAST search against the wheat genome database (IWGSC RefSeq v1.0) using rice and barley SOS1 sequences as queries. After the redundancy elimination and domain confirmation, the three sets of wheat *TaSOS1* homoeologs on chromosome 3AS, 3BS and 3DS were obtained and these homologous alleles were designated as *TaSOS1-A*, *TaSOS1-B*, or *TaSOS1-D* according to their chromosome location (**Figure 1A**). A sequence comparison between cDNA and genomic DNA revealed that *TaSOS1* has 23 exons and 22 introns (**Figure S1**). The coding sequences (CDS) of the three *TaSOS1* homoeologs were comparatively conserved, and can be distinguished from one another by series of single nucleotide polymorphisms (SNPs). In contrast with the CDS, differences in molecular size were found in introns and in upstream/downstream regions. Deduced amino acid sequence alignments showed that TaSOS1-A, TaSOS1-B and TaSOS1-D encode 1142 amino acid residues and three TaSOS1 homoeologs shared 98.16% sequence similarity. The amino acid sequences differed from one another at 49 positions (**Figure 1B**). In addition, similar to its orthologues from *Arabidopsis*, the predicted proteins of TaSOS1 homoeologs contain 12 transmembrane domains (TM) in N-terminus and all of them are conserved in three TaSOS1 homoeologs, except that a single amino acid substitution in the first predicted membrane-spanning region of TaSOS1-D (Ala-Gly). Previously analysis in *Arabidopsis* showed that AtSOS1 protein contains three pivotal and highly conserved consensus motifs, including CNBD activation domain in N-terminus, auto-inhibitory domain and phosphorylation site in C-terminus. The alignments revealed the phosphorylation site and the auto-inhibitory domain highly conserved at the C-terminus of three TaSOS1 homoeologs (**Figure 1B**). However, the sequence of CNBD activation domain, a conserved domain interacts with auto-inhibitory domain to keep the SOS1 protein with basal activity under control condition, show differences between TaSOS1-D and TaSOS1-A, TaSOS1-B (**Figure 1B**).

### Expression patterns of wheat *TaSOS1* in different tissues under salt stress

**Figure 2:**
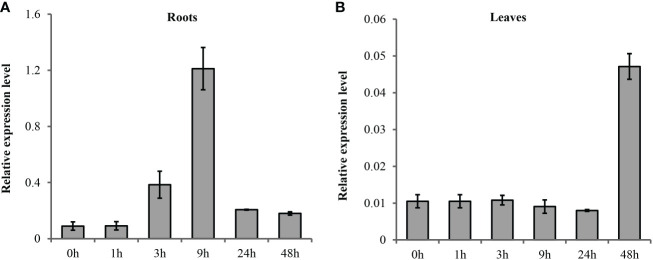
Quantitative RT-PCR analysis of the expression of *TaSOS1* genes in response to salt stress in wheat. Transcript levels of *TaSOS1* genes detected in 7-day-old wheat seedling roots **(A)** and leaves **(B)** were subjected to 0 mM or 200 mM NaCl treatment with indicated time course. The expression of *β-Actin* was used as an endogenous control. Error bars represent the standard deviations of three technical replicates, and the results were consistent in three biological replicates.

The spatial and temporal expression patterns of *TaSOS1* under normal and salt stress condition were first analyzed in a wheat variety Nongda3338 by quantitative real-time PCR (qRT-PCR). The results indicated the transcription abundance of *TaSOS1* were improved after salt treatment but the transcription patterns were different in seedling shoot and root. In root, the expression of *TaSOS1* was induced quickly and peaked at 9 h after salt treatment then the level of mRNA accumulation gradually declined (**Figure 2A**). However, the mRNA accumulation remains almost unchanged in shoot until 48h after salt condition (**Figure 2B**).

### Expression of wheat *TaSOS1* gene was correlated with salt tolerance variation in polyploidy wheat

**Figure 3:**
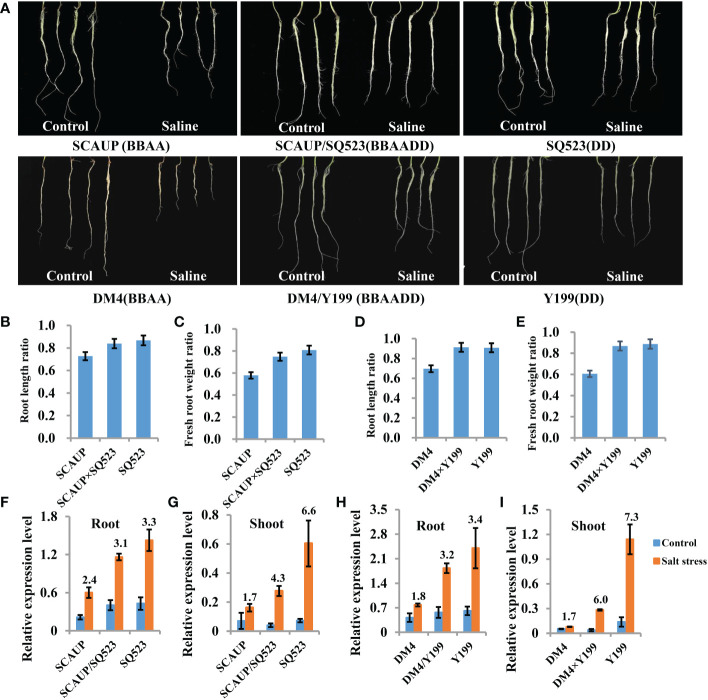
Salt stress phenotypes and transcript level of *SOS1* genes in synthetic allopolyploid wheats and their parents. Seedlings of the two set of synthetic hexaploids with their tetraploid and diploid parents were first grown for 10 d without NaCl treatment. The plants were then grown under control conditions (Control) or treated with 200 mM NaCl solution (Saline); phenotypes were measured and photographed after 7 d. SCAUP/SQ523 and DM4/Y199, synthetic allohexaploid wheats, BBAADD; SCAUP and DM4, tetraploid parents, BBAA; SQ523 and Y199, diploid parents, DD. **(A)** Root phenotypes of with tetraploid and diploid parents upon NaCl treatment. **(B-E)** Quantification of root length **(B, D)** and fresh weight **(C, E)** of two synthetic allotetraploid wheats and their tetraploid and diploid parents. Mean and SD values were derived from measurements of at least 20 seedlings from three independent assays. **(F-I)** qRT‐PCR analysis of *SOS1* genes in roots **(F, H)** and shoots **(G, I)** of two set of synthetic hexaploids with their tetraploid and diploid parents in response to salt stress. Seven-day-old seedling plants were subjected to 0 mM or 9h after 200 mM NaCl treatment for root or after 2 days of 200 mM NaCl treatment for shoot. The expression of *β-Actin* was used to normalize mRNA levels. The values are means (± SE) of three biological replicates.

**Figure 4:**
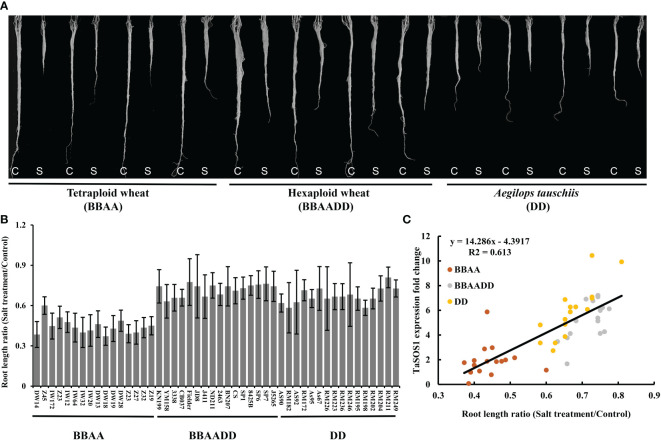
Expression of *SOS1* gene was correlated with salt tolerance variation in wheat accessions of different ploidy. **(A)** Example of root length phenotype of natural wheat accessions before and after 200 mM NaCl treatment. C: Control; S: Salt treatment. **(B)** A quantitative assessment of the roots length for 48 natural wheat accessions. The y-axis denotes a ratio of NaCl-treated seedlings relative to the same genotype grown on control condition. Mean and SD values were derived from measurements of at least 20 seedlings from three independent assays. **(C)** Correlation coefficients between the roots length ratio (Salt treatment/Control) and the expression levels of *TaSOS1* among different ploidy wheat accessions. The x-axis denotes a ratio of NaCl-treated seedlings relative to the same genotype grown on control condition. The y-axis denotes the fold change of *TaSOS1* up-regulation expression in root of wheat accessions with different ploidy before and after 200 mM NaCl treatment.

Hexaploid bread wheat has a greater discriminating capacity between Na^+^ and K^+^ than durum wheat under salt stress and is generally more salt tolerant. In order to assess the salt tolerance variation after the formation of allopolyploid wheat, two set of synthetic hexaploids (SCAUP/SQ523 and DM4/Y199, BBAADD) with their tetraploid (SCAUP and DM4, BBAA) and diploid *Ae. tauschii* (SQ523 and Y199, DD) parents were used for salt tolerance assessment. The root length, root and shoot fresh weight for each of the three genotypes under normal and salt stress conditions were recorded and quantified. Consistent with previous study (Zheng et al., 2021), the results indicated the synthetic hexaploid wheats and diploid *Ae. tauschii* exhibited superior growth vigour relative to the tetraploid lines (**Figures 3A-E**; **Figure S2**). We further analyzed the expression patterns of *TaSOS1* gene in two synthetic hexaploids with their tetraploid and diploid parents using qRT-PCR. Consistent with our expectation, the *TaSOS1* genes were up-regulated in synthetic hexaploid wheats (SCAUP/SQ523, DM4/Y199) compared with their tetraploid parents (SCAUP, DM4) both in seedling roots and shoots upon NaCl treatment. Notably, the expression of *TaSOS1* was upregulated more significantly in diploid (SQ523, Y199) parents (**Figures 3F-I**). Thus, we examined whether the expression pattern of *TaSOS1* is correlative to the variation in the salt tolerance (scored root length as a growth indicator and compared the changes before and after salt treatment) in 48 natural wheat accessions with varying ploidy, including 16 allohexaploid wheats, 16 allotetraploid and 16 diploid species *Ae. tauschiis* (**Figures 4A, B**; **Table S1**). Across the three different ploidy levels, the fold change of *TaSOS1* upregulation expression was positively correlated with the root length under salt treatment of the natural wheat accessions (BBAADD, BBAA and DD; R^2^ = 0.613, P < 0.01; **Figure 4C**). In addition, we also performed the correlation analysis between the *TaSOS1* expression pattern with the change of root and shoot fresh weight before and after salt treatment in 48 natural wheat accessions, these results also indicated the fold change of *TaSOS1* up-regulation expression was positively correlated with salt tolerance in wheat accessions of different ploidy (**Figures S2A-E**).

### Subgenome expression divergence of three *TaSOS1* homoeologs in hexaploid wheat

**Figure 5:**
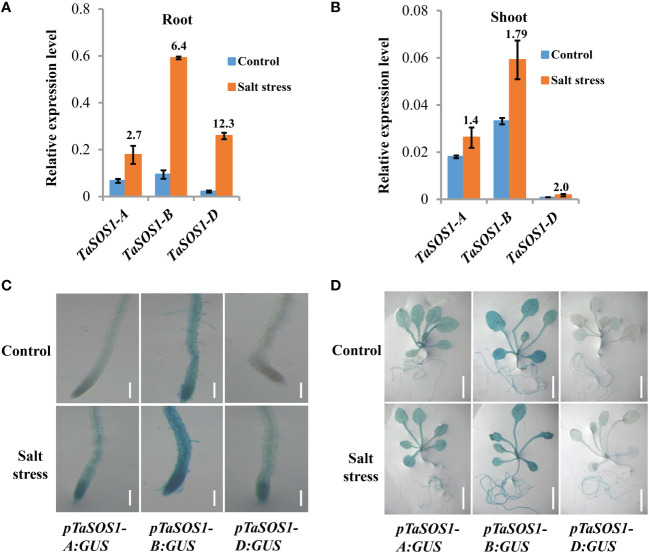
Expression patterns of three *TaSOS1* homoeologous genes in response to salt stress in allohexaploid wheat. **(A, B)** Transcript levels of three *TaSOS1* homoeologous genes in roots and shoots of allohexaploid wheat upon NaCl treatment. qRT-PCR analysis the expression of *TaSOS1-A*, *TaSOS1-B* and *TaSOS1-D* genes with gene-specific primers in wheat seedling roots that subjected to 9 h treatment with 200 mM NaCl or in wheat seedling leaves subjected to 2 d treatment with 200 mM NaCl. Fold change ratio was labeled above the bars. The expression of *β-Actin* was used to normalize mRNA levels. The values are means (± SE) of three biological replicates. **(C, D)** Spatial expression patterns of the *TaSOS1-A*, *TaSOS1-B* and *TaSOS1-D* genes in transgenic *Arabidopsis* plants harboring their promoters fused to the *GUS* gene, respectively. Promoter activity was visualized through histochemical GUS staining in roots **(C)** and seedlings plants **(D)** transferred to normal medium (control) or to medium containing 200 mM NaCl (salt stress) for 1 days. Scale bars: root, 200 µm; whole seedling plants, 1 cm.

To further investigate how the expressions of *TaSOS1* homoeologous genes were regulated in allopolyploid wheat under salt stress, gene-specific PCR primers were designed and the amplification efficiency of the primers was confirmed by homoeolog-specific genomic PCR (**Figure S3A**). Considering the expression of *TaSOS1* peaked at 9 h in root and 2 d after salt stress in shoot, we coupled quantitative RT-PCR to quantity the *TaSOS1-A*, *TaSOS1-B* and *TaSOS1-D* subgenome specific expression at these specific time points after salt stress in root and shoot, respectively. Interestingly, different expression patterns were found between the three *TaSOS1* homoeologous genes. In root, NaCl treatment increased the transcription level by more than 12 times of *TaSOS1-D* and 2.7-, 6.4-fold enhancements were observed of the amount of *TaSOS1-A* and *TaSOS1-B* (**Figure 5A**). However, in shoot, the amount of *TaSOS1-B* mRNA was much higher than *TaSOS1-A* and *TaSOS1-D*, while the transcription level of *TaSOS1-D* was the lowest in both control and salt condition (**Figure 5B**). Taken together, these results showed that these homoeologs may play versatile roles in different organs under salt stress condition.

To gain further insight into the transcriptional divergence of three *TaSOS1* homoeologous genes in normal and salt treatment conditions, we generated transgenic *Arabidopsis* plants expressing the *β-glucuronidase* (GUS) gene (*uidA*) under the control of the *TaSOS1-A*, *TaSOS1-B* and *TaSOS1-D* promoter, respectively. About 2 kb genomic DNA fragments which included the sequence upstream of transcription initiation site of three *TaSOS1* homoeologs were amplified, respectively. The reporter constructs using *uidA* fused to the promoter sequence of *TaSOS1-A*, *TaSOS1-B* and *TaSOS1-D* (*pTaSOS1-A:GUS*, *pTaSOS1-B:GUS*, *pTaSOS1-D:GUS*) were transformed into the wild type *Arabidopsis* (Columbia ecotype) (**Figure S3B**). The construct was introduced *via Agrobacterium*-mediated transformation by the floral-dip method. For each transgenic assay, three independent T_3_ homozygous lines carrying the construct for a single insertion were selected and used for further characterization (**Figure S3C**). The GUS activity of transgenic *Arabidopsis* plants carrying *pTaSOS1-A:GUS*, *pTaSOS1-B:GUS* and *pTaSOS1-D:GUS* were examined under normal or salt conditions by histochemical staining (**Figures 5C, D**). In normal conditions, the GUS activity can be detected in roots, stems and leaves of *pTaSOS1-A:GUS* and *pTaSOS1-B:GUS* seedling plants, but undetectable in leaves and slightly in root of *pTaSOS1-D:GUS* lines (**Figures 5C, D**). These differential expression pattern between the three promoters are consistent with our qRT-PCR results in wheat. To assess the expression of *TaSOS1s* under salt stress conditions, transgenic *Arabidopsis* seedlings were subjected to 200 mM NaCl treatment for 1 d, after which the seedlings were stained. The results showed that NaCl induced deeper blue staining in root of *pTaSOS1-B:GUS* than *pTaSOS1-A:GUS* and *pTaSOS1-D:GUS* lines, but the improvement ratio of GUS activity of *TaSOS1-D* in root was more than *TaSOS1-A* and *TaSOS1-B* (**Figure 5C**). Meanwhile, the histochemical staining showed that the activities of the three wheat *TaSOS1* promoters in leaves under salt stress almost unchanged or a bit higher than untreated controls (**Figure 5D**). Collectively, our results, on the one hand, were consistent with our qRT-PCR results showing that the transcription patterns of *TaSOS1-A*, *TaSOS1-B* and *TaSOS1-D* existed discrepancies under normal and salt treatment. On the other hand, the results indicated that *TaSOS1-A* and *TaSOS1-B* showed high basal expression in roots and leaves in normal condition and they can further up-regulation in roots under salt stress. While *TaSOS1-D* showed markedly lower expression in both roots and leaves under normal conditions, but showed significant up-regulation in roots but not leaves under salt stress.

### The *cis*-acting element analysis for promoters of three *TaSOS1* homoeologs

To explore the molecular basis of the differential expression pattern of three *TaSOS1* homoeologous genes, *cis*-acting elements analysis for promoters of three *TaSOS1* homoeologs was carried out through web-based PLANTCARE and PLACE databases (Lescot et al., 2002). Results showed that there are 23 cis-acting elements owned by all the three *TaSOS1* promoters and several of them involved in the activation of defense-related genes, including predicted ABREs, which are known to be involved in abscisic acid responsiveness. TGACG-motif, CGTCA-motif, which are involved in jasmonic acid methyl ester (MeJA) responses. AuxRR-core, which is associate with auxin responsiveness. Further, several elements involved in light responsiveness such as G-box, ATC-motif, and CAT-box, which is related to meristem expression were also present in the promoters of three *TaSOS1* homoeologs (**Figure S4**). Moreover, there are several types of *cis*-acting elements were detected only in one or two promoter regions of three *TaSOS1* genes, such as EIRE and O2-site motif only presented in *TaSOS1-A*; while ACE, ATCT-motif, CATT-motif, HSE and TC-rich repeats are presented both in promoters of *TaSOS1-A* and *TaSOS1-B*, but absent in *TaSOS1-D*. Interestingly, we also found the P-box, AP-2-like and WUN-motif are specifically exhibited in promoter of *TaSOS1-D.* These results suggest there are genetic variations in cis-regulatory elements or regulatory regions in the three *TaSOS1* homoeologs and may be involved in gene expression changes that impact phenotype.

### Three *TaSOS1* homoeologous genes show a different contribution to salt tolerance

**Figure 6:**
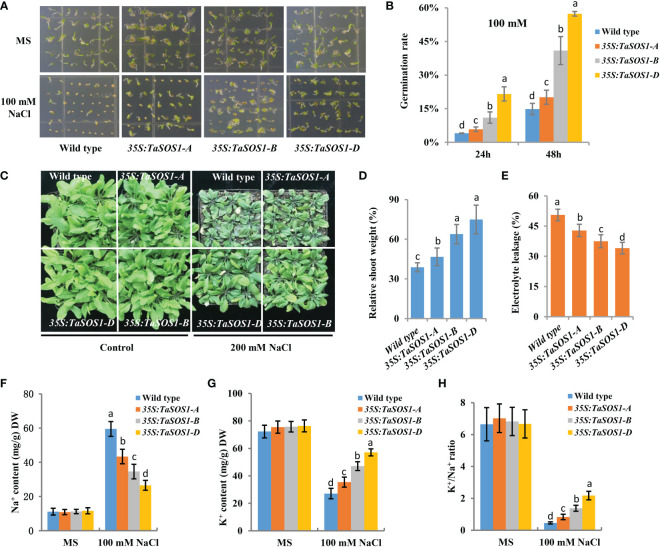
Phenotypic analysis of the *35S:TaSOS1-A, 35S:TaSOS1-B* and *35S:TaSOS1-D* plants exposed to salt stress. **(A, B)** Comparison of the seed germination of the wild type, the *35S:TaSOS1-A, 35S:TaSOS1-B* and *35S:TaSOS1-D* plants. Seeds harvested on the same day were planted on MS medium containing 0, or 100 mM NaCl. Plates were transferred to a growth chamber after stratification at 4°C for 3 d. Photographs were taken, and germination rate was determined 24 h and 48 h after the transfer, respectively. **(C, D)** Comparison of seedlings growth of the wild type, the *35S:TaSOS1-A, 35S:TaSOS1-B* and *35S:TaSOS1-D* plants with 200 mM NaCl. Five-day-old seedlings were transferred from MS medium to soil. The plants were then irrigated under well-watered conditions (Control) or irrigated every 5 d with 200 mM NaCl solution; 15 d later, phenotypes were measured and photographed. Shoots fresh weight was measured relative to the same genotype grown on control condition. More than 30 plants were measured for each genotype. **(E)** Relative electrolyte leakage of leaves from wild type, *35S:TaSOS1-A, 35S:TaSOS1-B* and *35S:TaSOS1-D* plants after exposure to 200 mM NaCl. **(F-H)** Comparison of Na^+^ contents **(F)**, K^+^ contents **(G)**, and K^+^ to Na^+^ ratios **(H)** of leaves from *35S:TaSOS1-A, 35S:TaSOS1-B* and *35S:TaSOS1-D*, and wild type plants under salt stress conditions. The values are means ( ± SE) of three biological replicates. The data were analyzed by ANOVA one-way comparison followed by LSD test. Different letters above the bars indicate a significant difference at P < 0.05.

**Figure 7:**
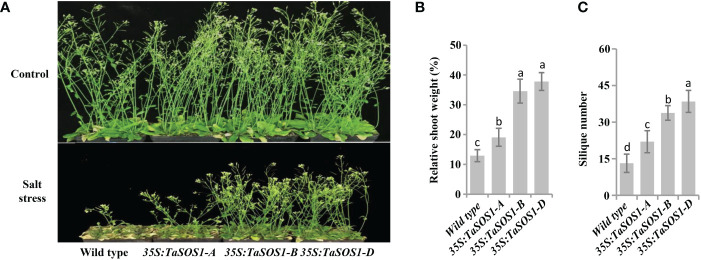
Comparison of 5-week-old of the wild type, the *35S:TaSOS1-A, 35S:TaSOS1-B* and *35S:TaSOS1-D* plants treated with 200 mM NaCl. **(A)** Five-day-old seedlings were transferred from MS medium to soil. The plants were then irrigated under well-watered conditions (Control) or irrigated every 5 d with 200 mM NaCl solution; 30 d later, phenotypes were measured and photographed. **(B)** and **(C)** Shoots fresh weight and silique number were measured relative to the same genotype grown on control condition. More than 24 plants were measured for each genotype. The values are means ( ± SE) of three biological replicates. The data were analyzed by ANOVA one-way comparison followed by LSD test. Different letters above the bars indicate a significant difference at P < 0.05.

To further understand the functions of three *TaSOS1* homoeologs in wheat, ectopic expression of *TaSOS1-A*, *TaSOS1-B* and *TaSOS1-D* were performed using *Arabidopsis* transgenic system. For each transgenic line, three independent T_3_ homozygous overexpression lines carrying the construct for a single insertion were selected and used for further characterization. RT-PCR analysis showed that the *TaSOS1* were expressed in all the transgenic *Arabidopsis* plants, respectively, but was absent in the wild-type (**Figure S5A**). We investigated the effects of three *TaSOS1* homoeologs overexpression on the morphology and growth of transgenic plants under normal and salt conditions. Germination rates of *TaSOS1*-overexpressing and wild type *Arabidopsis* seeds were first compared. In normal conditions, the transgenic lines of *35S:TaSOS1-A*, *35S:TaSOS1-B* and *35S:TaSOS1-D* and wild type plants showed similar germination rates (**Figure 6A**). In MS medium with 100 mM NaCl, three *TaSOS1* homoeologs transgenic lines exhibited higher germination rates than wild type plants (**Figure 6A**). Notably, the transgenic lines of *35S:TaSOS1-D* displayed more salt tolerant phenotype than *35S:TaSOS1-A* and *35S:TaSOS1-B* plants. The germination rate for *35S:TaSOS1-D* lines were nearly 60% in 48h under salt stress treatment, but only 20% for *35S:TaSOS1-A* and 41% for *35S:TaSOS1-B* lines during the same time period (**Figure 6B**). More significantly, when sowed in MS medium with 200 mM NaCl, the seed germination of *35S:TaSOS1-D* transgenic lines were significantly higher than that of the *35S:TaSOS1-A*, *35S:TaSOS1-B* and wild type plants, with more than 30% of the seeds of the *35S:TaSOS1-D* transgenic lines germinating in the 84h after sowing, while less 6% of the *35S:TaSOS1-A*, *35S:TaSOS1-B* transgenic lines and wild type seeds germinated at the same treatment (**Figures S5B, C**). We further determined the seedlings growth of the wild type and the three *TaSOS1* homoeologs transgenic plants and found that the growth performance of transgenic lines was similar to that of wild type plants when grown in soil pots in non-saline conditions (**Figure 6C**). When irrigated with 200 mM NaCl for 15 d, three *TaSOS1* homoeologs transgenic lines exhibited a salt tolerant phenotype compared with wild type plants (**Figure 6C**). We scored the shoot fresh weight and compared the changes between transgenic and wild type plants before and after salt treatment. Overall, NaCl had a lower inhibitory effect on *TaSOS1* overexpression lines than it had on the wild type plants. The shoot fresh weight of wild type was 38% of the control weight, while the shoot fresh weight of *TaSOS1-B* and *TaSOS1-D* overexpression lines was more than 70% of the control weight (**Figure 6D**). We also determined the relative electrolyte leakage (REL) of wild type, *35S:TaSOS1-A*, *35S:TaSOS1-B* and *35S:TaSOS1-D* plants after salt stress and found that the REL of the *TaSOS1-*overexpression lines was significantly lower than that of the wild type (**Figure 6E**). Since low Na^+^ content and high K^+^/Na^+^ ratio in cells are crucial for salt tolerance, we further compared the Na^+^, K^+^ content and K^+^/Na^+^ ratio in leaves of three *TaSOS1* homoeologs transgenic lines and wild type under salt stress conditions. As shown in **Figures 6F-H**, generally, the *TaSOS1* overexpression lines keep less Na^+^, more K^+^ content and higher K^+^/Na^+^ ratio than wild type and among them, *35S:TaSOS1-D* showed lower Na^+^ content and higher K^+^/Na^+^ than the *35S:TaSOS1-A*, *35S:TaSOS1-B* transgenic lines. Furthermore, all *TaSOS1* homoeologs transgenic lines exhibited a salt tolerant phenotype compared with wild type plants in the reproductive growth stage. Among them, *35S:TaSOS1-B* and *35S:TaSOS1-D* showed much less growth retardation than the *35S:TaSOS1-A* transgenic lines and wild type plants, including fresh weight, silique number and plant height (**Figures 7A-C**; **Figure S5D**). Thus, the results demonstrate that three *TaSOS1* homoeologous genes display different degree of contribution to salt tolerance at different developmental stages and among of them, *TaSOS1-A* contributes the least to salt tolerance in all developmental stages, while *TaSOS1-D* plays the prominent role in germination and seedling stage and both *TaSOS1-B* and *TaSOS1-D* exhibit a higher contribution in adult stage under salt stress.

## Discussion

The majority of polyploid crops, including wheat, cotton and rape, originated by hybridization between different species. Allopolyploidy results in the convergence of different genomes previously adapted to different environments, thus often exhibit increased vigor and tend to have broader geographic distributions than those of their close diploid relatives (Dubcovsky and Dvorak, 2007; Feldman et al., 2012; Pallotta et al., 2014; Bird et al., 2018). In this study, we determined the phenotypes and measured physiological indexes of two set of synthetic allohexaploid wheat with their parents and 48 natural wheat accessions with different ploidy levels under normal and salt condition. We show that the both the natural and synthetic hexaploid wheat and diploid *Ae. tauschii* exhibit greater salinity tolerance than tetraploid wheat. This was supported by our observations that for most of the tested phenotype and physiological indexes directly related to salt tolerance, such as growth vigour, relative shoot and root fresh weight, root length, were highly similar between the synthetic hexaploid wheats and diploid parents *Ae. tauschii*, but substantially different from their tetraploid progenitor species (**Figures 3**, **4** and **Figure S2**). These results indicated that tetraploid wheats are relatively sensitive to high salinity and allohexaploid wheats and diploid *Ae. tauschii* have a higher fitness under salt stress, which was in line with the reports before (Yang et al., 2014; Zheng et al., 2021). The mechanisms behind this trait has always been a topic of great interest to scientists. Previous studies indicated that one of the major contributors behind this difference is that hexaploid wheats maintain a higher K^+^/Na^+^ ratios in the leaves (Munns and Tester, 2008; Wang and Xia, 2018), and genetic analysis demonstrated this trait was controlled by *Kna1* locus on chromosome 4D (Dvorak et al., 1994; Dubcovsky et al., 1996). Further studies indicated *TaHKT1;5-D* should be the candidate gene of *Kna1* (Huang et al., 2008; Byrt et al., 2014). In the present study, we found there is significant positive correlation between the transcriptional alteration of *TaSOS1* and the variation in the salt tolerance of different wheat species, suggesting its important role in the regulation of polyploidy wheat salt tolerance (**Figures 3**, **4** and **Figure S2**). Furthermore, both qRT-PCR and histochemical GUS staining results showed that there exist subgenome bias of *SOS1* expression in hexaploid wheat. Among of three *TaSOS1* homoeologs, *TaSOS1-A* and *TaSOS1-B* showed high basal expression in roots and leaves in normal conditions and further up-regulated in roots under salt stress. While *TaSOS1-D* showed markedly lower expression both in roots and leaves under normal conditions, but significant up-regulated in roots but not leaves under salt stress (**Figure 5**). It appears to be the rule that allopolyploidization induces rapid and dynamic changes of genome and chromatin structures, as well as reorchestration of homeologous gene expression, which may represent a major advantage of allopolyploidy over its progenitors (Ding and Chen, 2018). Yang et al. found that *TaHKT1;5-D* exhibited a transcriptional reprogramming from constitutive high basal expression in diploid *Ae. tauschii* to salt-induced expression in a newly synthetic allohexaploid wheat and natural hexaploid bread wheat (Yang et al., 2014). In this respect, our results are consistent with the previous findings that suggested condition-dependent gene expression reorchestration of the subgenomes might have contributed to the wide-ranging adaptability of natural allopolyploidy plants (Roulin et al., 2013; Powell et al., 2017). It is generally believed the genetic variations in regulatory regions or cis-regulatory elements may lead to gene expression changes that impact phenotype and these variations can be a potential driving force of evolution, creating genetic diversity that can be used for domestication and breeding innovation (Swinnen et al., 2016; Springer et al., 2019). We also found that several types of *cis*-acting elements were detected only in one or two promoter regions of three *TaSOS1* homoeologs (**Figure S4**). Further research on these three *TaSOS1* homoeologs expression divergence, such as identifying the other key element in promoters will be meaningful to reveal the regulation mechanisms. Moreover, a growing body of research suggests that epigenetic modifications contribute to the expression modulation of homoeologs in allopolyploid species (Shitsukawa et al., 2007; Hu et al., 2013; Bird et al., 2018; Ding and Chen, 2018), so it is worth to dissect the possible epigenetic mechanisms of the expression divergence in *TaSOS1* homoeologs in wheat.

It has been shown that subgenomes of a given allohexaploid species have redundant but also distinct functions, which may represent a major advantage of allopolyploidy over its diploid progenitors (Shitsukawa et al., 2007; Yang et al., 2014; Uauy, 2017). Notably, we found that three *TaSOS1* homoeologous genes showed a differential contribution to salt tolerance at different developmental stages. This was supported by our observations that for most of the measured morphophysiological traits directly related to salt tolerance, including germination rate, growth vigor, osmotic regulation and Na^+^, K^+^ content and K^+^/Na^+^ ratio, were greater in *35S:TaSOS1-D* transgenic lines than that of the *35S:TaSOS1-A*, *35S:TaSOS1-B* and wild type plants in germination and seedling stage, while both *TaSOS1-B* and *TaSOS1-D* exhibit more prominent contribution than *TaSOS1-A* and wild type plants in adult stage under salt stress condition. Therefore, we speculated the enhancement in salt tolerance of hexaploid wheat maybe partly due to the of *TaSOS1-D* contribution. This is consistent with the findings of previous study, which demonstrated that to a great extent the salt-tolerant trait of hexaploid wheat can be attributed to the D genome by allopolyploidization (Byrt et al., 2014; Zheng et al., 2021). Nevertheless, in allohexaploid wheat, we found the transcriptional level of *TaSOS1-D* was suppressed in shoot and mainly induced in root when treated with salt stress. One possible explanation is that the enhanced expression of *TaSOS1-D* in the root is adequate for extruding Na^+^ to the external soil environment and provides the first barrier to Na^+^ uptake in hexaploid wheat (Katschnig et al., 2015; Assaha et al., 2017).

It has been reported that SOS1 encode an over 120-kDa protein with 12 transmembrane domains in the N-terminal part and a long hydrophilic cytoplasmic tail in the C-terminal part (Shi et al., 2000). SOS1 is maintained in resting state through the intra-molecularly interaction of the auto-inhibitory domain with an adjacent domain of SOS1 in the absence of stress (Quintero et al., 2011). Previous study showed that deletion of the auto-inhibitory domains of *TdSOS1* conveyed much greater salt tolerance to the transgenic *Arabidopsis* (Feki et al., 2014). In our study, amino acid sequence alignment showed that both the auto-inhibitory domains and adjacent motif of three TaSOS1 homoeologs were highly conserved (**Figure 1B**), suggesting these domains maybe not involved in the functional divergence of TaSOS1 homoeologs under salt stress. Research findings in *Arabidopsis* indicated the activation domain CNBD of SOS1 can interact with its auto-inhibitory domain to keep the transporter in a resting state with basal activity (Shi et al., 2000). The putative CNBDs of the TaSOS1-D is different with TaSOS1-A and TaSOS1-B proteins. There is a leucine (L) at the residue 513 in the TaSOS1-A and TaSOS1-B proteins but an isoleucine (I) in the TaSOS1-D at the same site (**Figure 1B**). The amino acid difference in the TaSOS1-D protein may create a critical turn in the polypeptide secondary structure and change the interaction between the putative CNBDs and auto-inhibitory domain, thereby alleviating TaSOS1-D auto-inhibition. Moreover, the transmembrane domains in the N-terminal part of SOS1 are critical for SOS1 function in plant salt tolerance. *Arabidopsis* mutants *sos1-3* (R-C), *sos1-12* (G-E), which contain single amino acid substitutions in the membrane-spanning region, probably abolish SOS1 antiporter activity (Shi et al., 2000). Protein structure prediction by TMHMM showed that all the three TaSOS1 proteins have the 12 transmembrane domains but there is a single amino acid difference in the first transmembrane domain between TaSOS1-D with TaSOS1-A and TaSOS1-B (**Figure 1B**). Collectively, we speculated that the amino acid variety maybe have an influence on the antiporter activity, hence TaSOS1-D may have an enhanced capacity in Na^+^ efflux and salt tolerance in transgenic lines. In addition, there are several amino acid differences at the other sites, such as at the first six amino acids in the N-terminus, but the effects of these differences are not clear, which need further investigation. Moreover, it must be noted that the heterologous expression of *TaSOS1* in *Arabidopsis* may not fully state the function of *TaSOS1* in wheat, further studies are required to explore the condition-dependent subfunctionalization of three *TaSOS1* homoeologs in wheat and assess specificity as well as generality of the phenomenon in response to abiotic stress. Taken together, our studies suggest that re-orchestration of homeologous genes expression and subfunctionalization of the plasma membrane Na^+^/H^+^ transporter *TaSOS1s* plays a key role of salt tolerance in hexaploid wheat. These results have provided insights into the subgenomic homoeologs variation potential to broad adaptability of natural polyploidy plant in wheat, which might effective for genetic improvement of salinity tolerance in wheat and other crops.

## Data availability statement

The original contributions presented in the study are included in the article/**Supplementary Material**. Further inquiries can be directed to the corresponding author.

## Author contributions

ZH and QS conceived the original screening and research plans. MZ, JPL, CZ, XL, WC, JCL, FW, WW, WG, MX, YY, HP and ZN performed the experiments and interpreted the data. ZN contributed to materials and methods. MZ and ZH conceived the project and wrote the article with contributions of all the authors. ZH agrees to serve as the author responsible for contact and ensures communication. All authors contributed to the article and approved the submitted version.

## Supplementary material

Supplementary Figure 1 Alignment of genomic and their corresponding cDNA sequences of three *TaSOS1* homoeologous genes.

Supplementary Figure 2 Expression of *SOS1* gene was correlated with salt tolerance variation in wheat accessions of different ploidy. **(A)** Example of aboveground phenotype of natural wheat accessions before and after 200 mM NaCl treatment. A quantitative assessment of shoots fresh weight **(B)** or roots fresh weight **(D)** for 48 natural wheat accessions. The y-axis denotes a ratio of NaCl-treated seedlings relative to the same genotype grown on control condition. Mean and SD values were derived from measurements of at least 15 seedlings from three independent assays. Correlation coefficients between the shoots fresh weight ratio **(C)** or roots fresh weight ratio **(E)** and the expression levels of *TaSOS1* among different ploidy wheat accessions. The x-axis denotes a ratio of NaCl-treated seedlings relative to the same genotype grown on control condition. The y-axis denotes the fold change of *TaSOS1* upregulation expression in shoot **(C)** or root **(E)** of wheat accessions with different ploidy before and after 200 mM NaCl treatment.

Supplementary Figure 3
**(A)** The amplification efficiencies of the gene-specific primers were tested by amplification of wheat genotype Nongda3338 genomic DNA. **(B)** Schematic of the *pTaSOS1-A:uidA*, *pTaSOS1-B:uidA* and *pTaSOS1-D:uidA* reporters. **(C)** The *pTaSOS1-A:uidA*, *pTaSOS1-B:uidA* and *pTaSOS1-D:uidA* transgenic lines confirmed by genomic PCR.

Supplementary Figure 4 The cis-acting regulatory elements analysis for promoter sequences of *TaSOS1-A*, *TaSOS1-B*, *TaSOS1-D* genes from hexaploid wheat genotype Nongda3338 using PlantCARE database. Red boxes: presented both in promoters of *TaSOS1-A* and *TaSOS1-B*, but absent in *TaSOS1-D*. Black boxes: presented specifically in promoter of *TaSOS1-A*. Blue boxes: presented specifically in promoter of *TaSOS1-D*.

Supplementary Figure 5 (A) The *35S:TaSOS1-A*, *35S:TaSOS1-B* and *35S:TaSOS1-D* transgenic lines confirmed by RT-PCR. (B) and (C) Comparison of the seed germination of the wild type, the *35S:TaSOS1-A, 35S:TaSOS1-B* and *35S:TaSOS1-D* plants on MS medium containing 0, or 200 mM NaCl. Seeds harvested on the same day were planted on MS medium. Plates were transferred to a growth chamber after stratification at 4°C for 3 d. Germination rate was determined every 24 h and photographs were taken at 5 d after the transfer. (D) Comparison the shoots height of 5-week-old of the wild type, the *35S:TaSOS1-A*, *35S:TaSOS1-B* and *35S:TaSOS1-D* plants growth with 200 mM NaCl. Shoots height were measured relative to the same genotype grown on control condition. More than 24 plants were measured for each genotype. The values are means ( ± SE) of three biological replicates. The data were analyzed by ANOVA one-way comparison followed by LSD test. Different letters above the bars indicate a significant difference at P < 0.05.
