# Supplementary material for: Subgenome-biased expression and functional diversification of a Na+/H+ antiporter homoeologs in salt tolerance of polyploid wheat
Source: Front Plant Sci. 2022 Dec 7;13:1072009. doi: 10.3389/fpls.2022.1072009 (PMC9768589; doi:10.3389/fpls.2022.1072009)
Supplement: Supplementary Figure 1 — Alignment of genomic and their corresponding cDNA sequences of three TaSOS1 homoeologous genes. [file DataSheet_1.pdf]

Figure S1

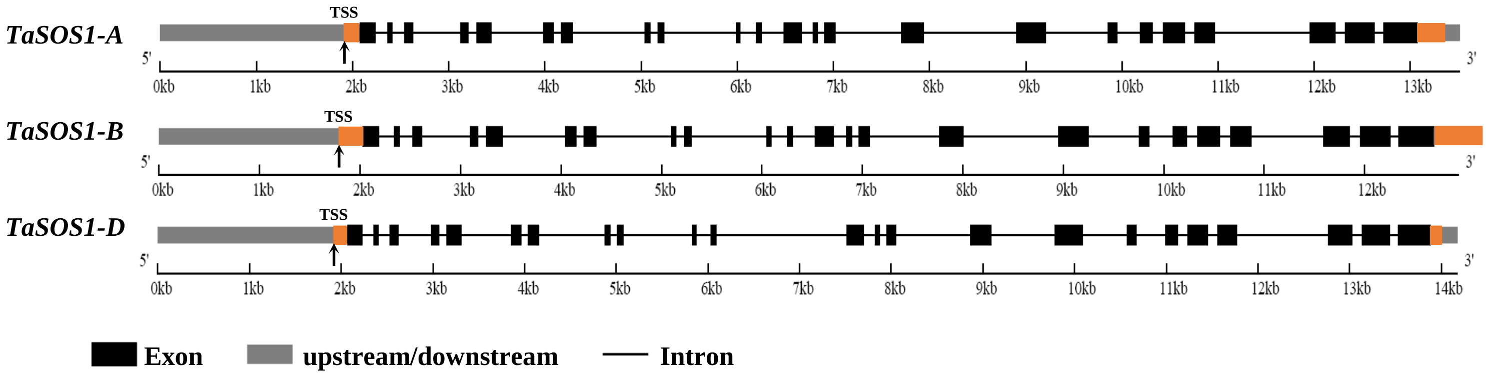
TaSOS1-A
TSS
5'
3'
0kb 1kb 2kb 3kb 4kb 5kb 6kb 7kb 8kb 9kb 10kb 11kb 12kb 13kb
TaSOS1-B
TSS
5'
3'
0kb 1kb 2kb 3kb 4kb 5kb 6kb 7kb 8kb 9kb 10kb 11kb 12kb
TaSOS1-D
TSS
5'
3'
0kb 1kb 2kb 3kb 4kb 5kb 6kb 7kb 8kb 9kb 10kb 11kb 12kb 13kb 14kb
Exon
upstream/downstream
Intron

Figure S2

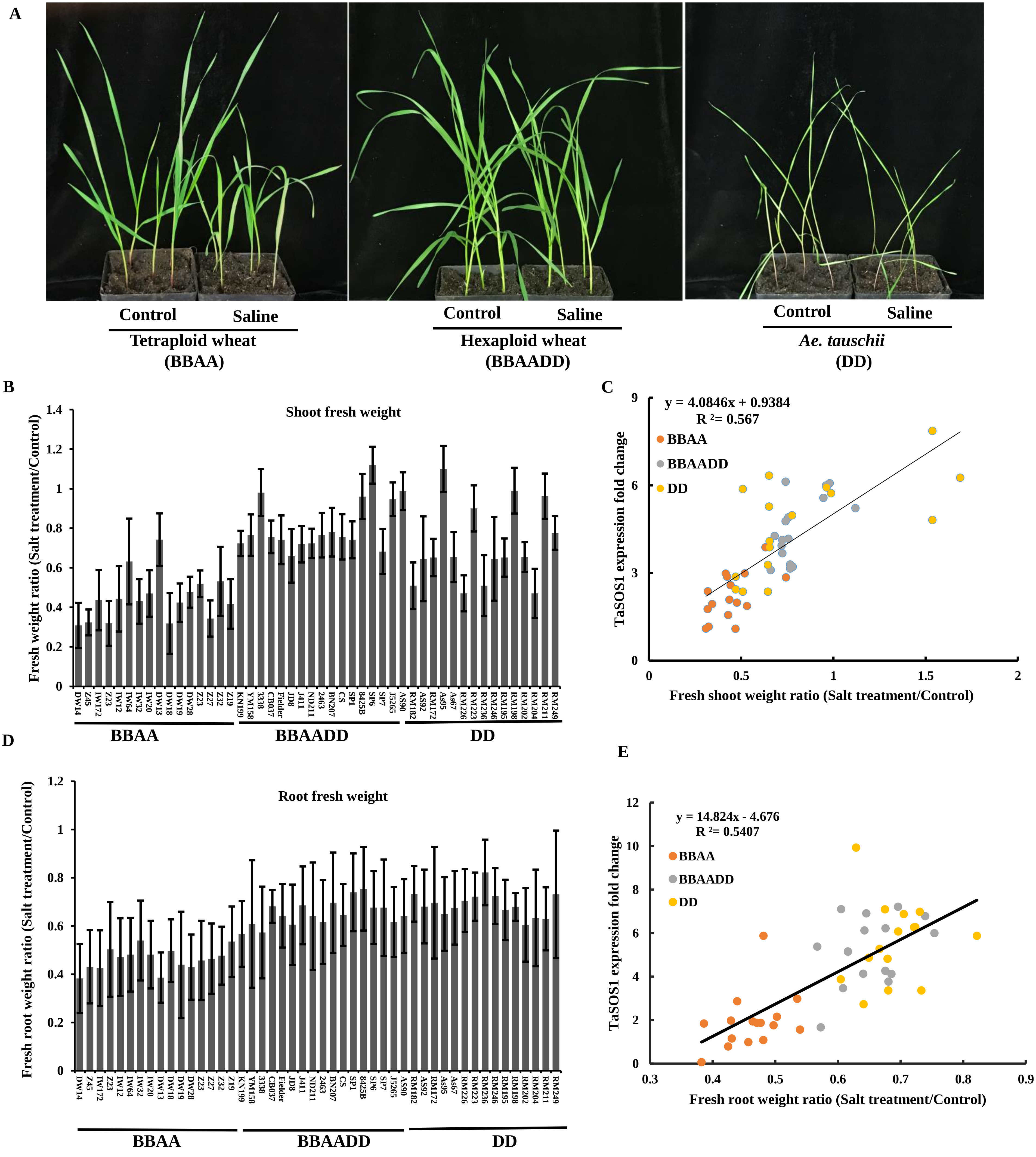
A
Control
Saline
Tetraploid wheat
(BBAA)
Control
Saline
Hexaploid wheat
(BBAADD)
Control
Saline
Ae. tauschii
(DD)
B
Shoot fresh weight
Fresh weight ratio (Salt treatment/Control)
0
0.2
0.4
0.6
0.8
1
1.2
1.4
DW14 Z45 IW172 Z23 IW12 IW64 IW32 IW20 DW13 DW18 DW19 DW28 Z23 Z27 Z32 Z19 KN199 YM158 3338 CB037 Fielder JD8 J411 ND211 2463 BN207 CS SP1 8425B SP6 SP7 J5265 AS90 RM182 AS92 RM172 As95 As67 RM226 RM223 RM236 RM246 RM195 RM198 RM202 RM204 RM211 RM249
BBAA
BBAADD
DD
C
y = 4.0846x + 0.9384
R^2= 0.567
BBAA
BBAADD
DD
TaSOS1 expression fold change
0
3
6
9
0
0.5
1
1.5
2
Fresh shoot weight ratio (Salt treatment/Control)
D
Root fresh weight
Fresh root weight ratio (Salt treatment/Control)
0
0.2
0.4
0.6
0.8
1
1.2
DW14 Z45 IW172 Z23 IW12 IW64 IW32 IW20 DW13 DW18 DW19 DW28 Z23 Z27 Z32 Z19 KN199 YM158 3338 CB037 Fielder JD8 J411 ND211 2463 BN207 CS SP1 8425B SP6 SP7 J5265 AS90 RM182 AS92 RM172 As95 As67 RM226 RM223 RM236 RM246 RM195 RM198 RM202 RM204 RM211 RM249
BBAA
BBAADD
DD
E
y = 14.824x - 4.676
R^2= 0.5407
BBAA
BBAADD
DD
TaSOS1 expression fold change
0
2
4
6
8
10
12
0.3
0.4
0.5
0.6
0.7
0.8
0.9
Fresh root weight ratio (Salt treatment/Control)

Figure S3

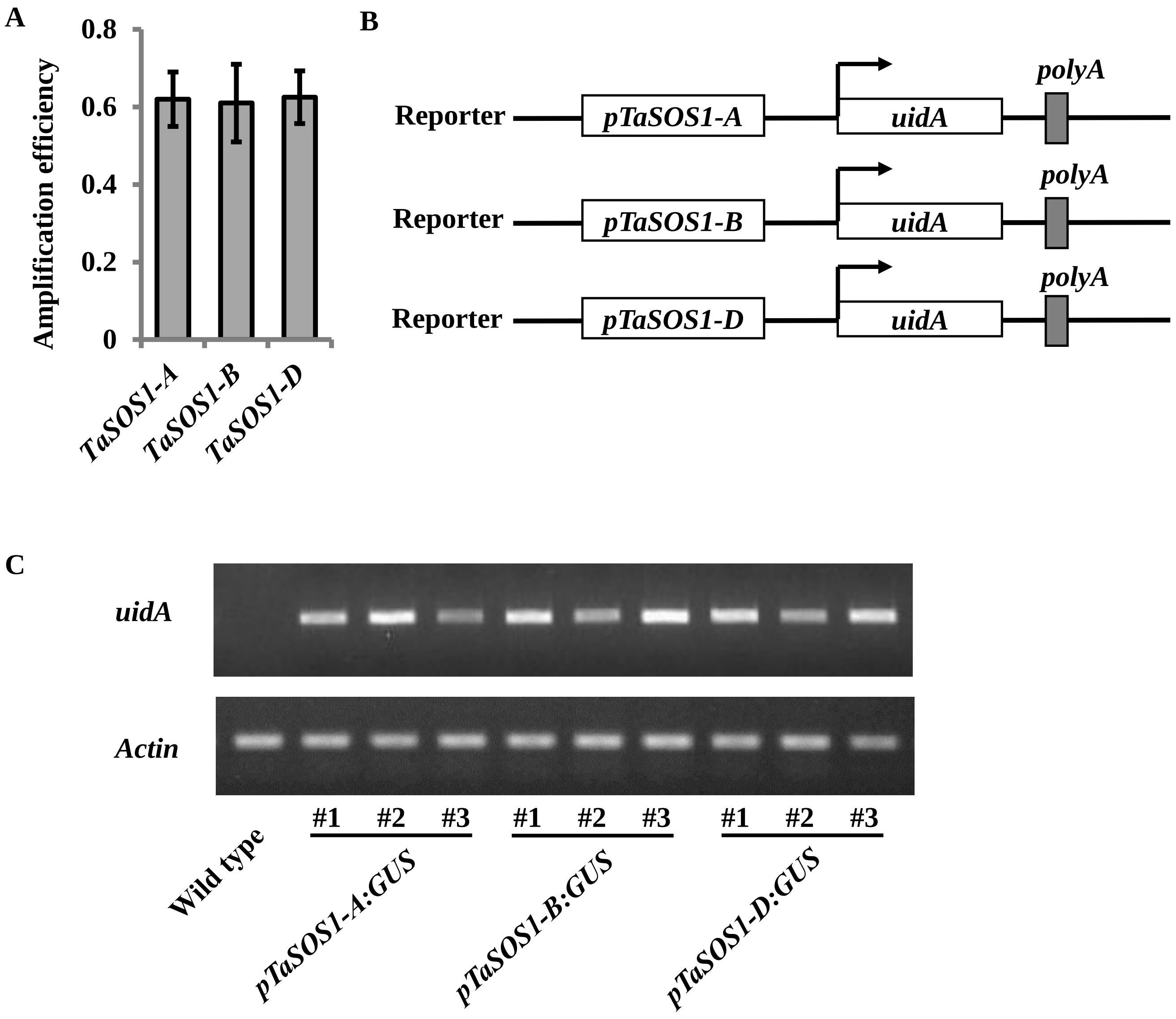

A
Amplification efficiency
0.8
0.6
0.4
0.2
0
TaSOS1-A
TaSOS1-B
TaSOS1-D
B
Reporter
pTaSOS1-A
uidA
polyA
Reporter
pTaSOS1-B
uidA
polyA
Reporter
pTaSOS1-D
uidA
polyA
C
uidA
Actin
#1 #2 #3
#1 #2 #3
#1 #2 #3
Wild type
pTaSOS1-A:GUS
pTaSOS1-B:GUS
pTaSOS1-D:GUS

Figure S4

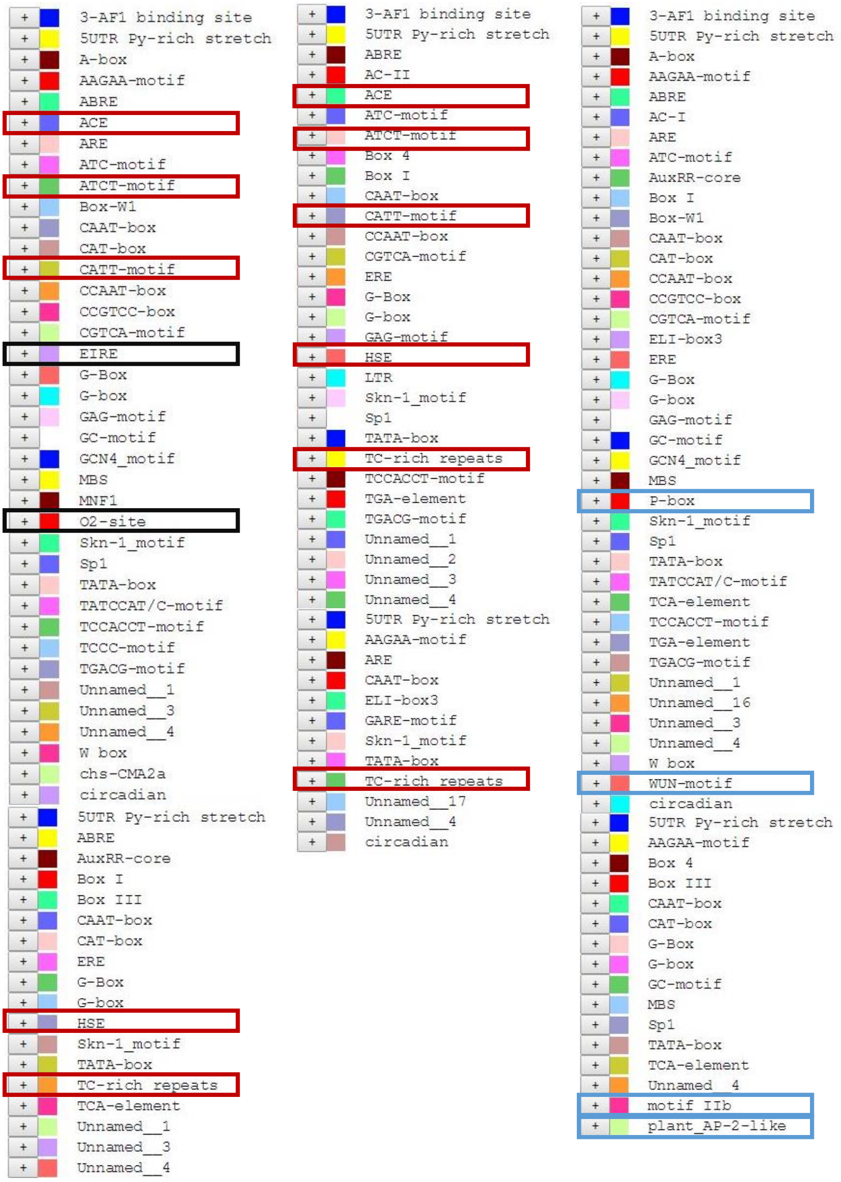

TaSOS1-A ***TaSOS1-B*** ***TaSOS1-D***

Figure S5

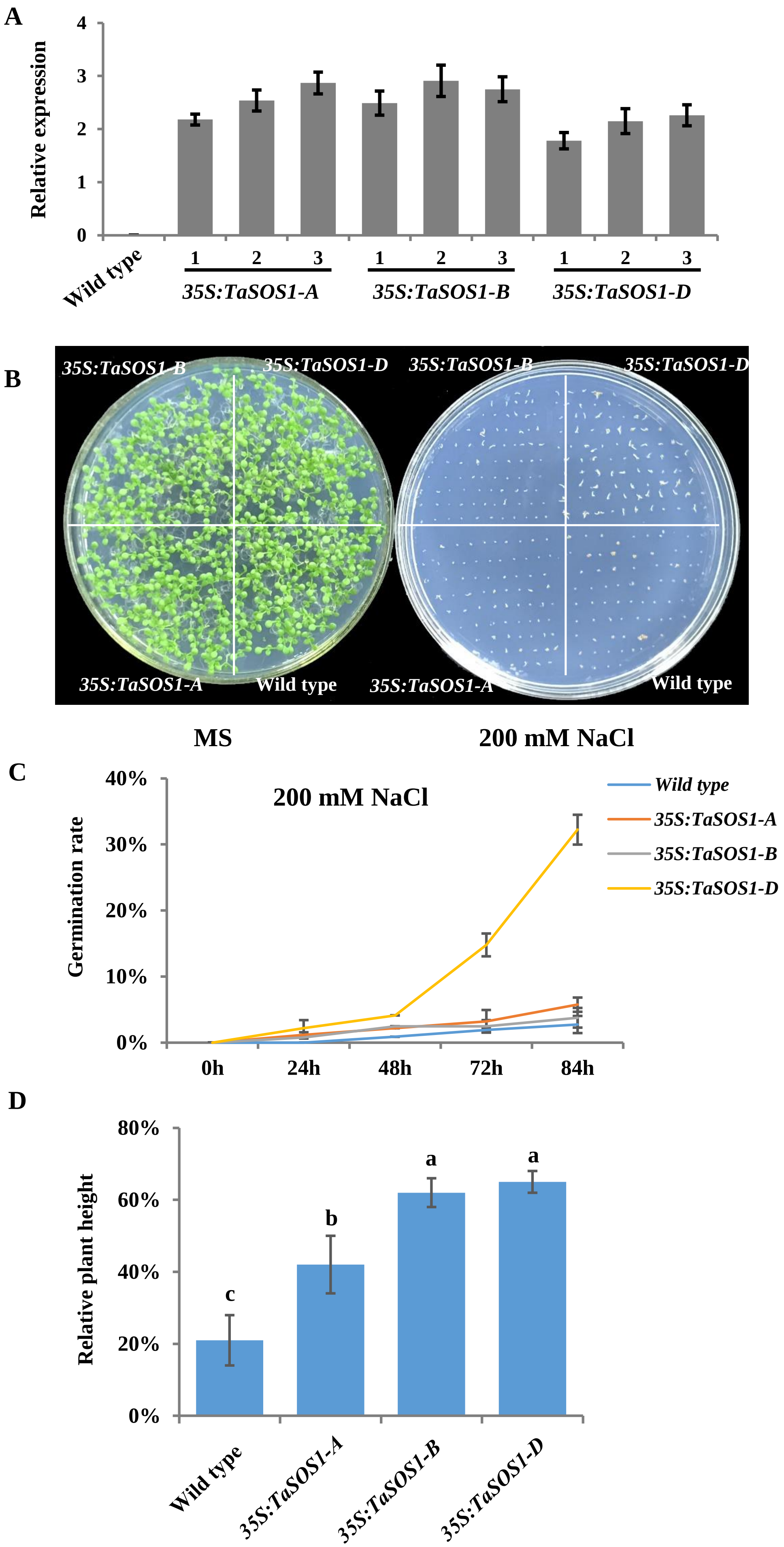
A
Relative expression
0
1
2
3
4
Wild type
1
2
3
35S:TaSOS1-A
35S:TaSOS1-B
35S:TaSOS1-D
B
35S:TaSOS1-B
35S:TaSOS1-D
35S:TaSOS1-A
Wild type
MS
200 mM NaCl
C
200 mM NaCl
Germination rate
0%
10%
20%
30%
40%
0h
24h
48h
72h
84h
Wild type
35S:TaSOS1-A
35S:TaSOS1-B
35S:TaSOS1-D
D
Relative plant height
0%
20%
40%
60%
80%
c
b
a
a
Wild type
35S:TaSOS1-A
35S:TaSOS1-B
35S:TaSOS1-D
